# Contribution of Nano-Zero-Valent Iron and Arbuscular Mycorrhizal Fungi to Phytoremediation of Heavy Metal-Contaminated Soil

**DOI:** 10.3390/nano11051264

**Published:** 2021-05-11

**Authors:** Peng Cheng, Shuqi Zhang, Quanlong Wang, Xueying Feng, Shuwu Zhang, Yuhuan Sun, Fayuan Wang

**Affiliations:** College of Environment and Safety Engineering, Qingdao University of Science and Technology, Qingdao 266042, China; cp1995a@163.com (P.C.); zsq9629@163.com (S.Z.); wql18764927102@163.com (Q.W.); fxy947320548@163.com (X.F.); zhangshuwu@126.com (S.Z.); yhsun@qust.edu.cn (Y.S.)

**Keywords:** heavy metals, nanoremediation, arbuscular mycorrhizal fungus, phytoremediation

## Abstract

Soil pollution with heavy metals has attracted increasing concern, which calls for the development of new remediation strategies. The combination of physical, chemical, and biological techniques can achieve more efficient remediation. However, few studies have focused on whether nanomaterials and beneficial microbes can be jointly used to facilitate phytoremediation. Therefore, we studied the role of nano-zero-valent iron (nZVI) and arbuscular mycorrhizal (AM) fungi in the phytoremediation of an acidic soil polluted with Cd, Pb and Zn, using sweet sorghum. X-ray diffraction (XRD), energy dispersive X-ray spectroscopy (EDS), and mapping analyses were conducted to explore the mechanisms of metal immobilization by nZVI. The results showed that although both bare nZVI (B-nZVI) and starch-stabilized nZVI (S-nZVI) inhibited root mycorrhizal colonization, *Acaulospora mellea* ZZ successfully colonized the plant roots. AM inoculation significantly reduced the concentrations of DTPA-Cd, -Pb, and -Zn in soil, and the concentrations of Cd, Pb, and Zn in plants, indicating that AM fungi substantially facilitated heavy metal immobilization. Both B-nZVI and S-nZVI, ranging from 50 mg/kg to 1000 mg/kg, did not impede plant growth, and generally enhanced the phytoextraction of heavy metals. XRD, EDS and mapping analyses showed that S-nZVI was more susceptible to oxidation than B-nZVI, and thus had more effective immobilization effects on heavy metals. Low concentrations of nZVI (e.g., 100 mg/kg) and AM inoculation had synergistic effects on heavy metal immobilization, reducing the concentrations of Pb and Cd in roots and enhancing root Zn accumulation. In conclusion, our results showed that AM inoculation was effective in immobilizing heavy metals, whereas nZVI had a low phytotoxicity, and they could jointly contribute to the phytoremediation of heavy metal-contaminated soils with sweet sorghum.

## 1. Introduction

The concentrations of heavy metals in soil have increased sharply in the past three decades, posing potential risks for the environment and human health [1]. The main human-made sources of soil heavy-metal(loid) pollution include agriculture, industry, mining, smelting, and waste disposal [1]. Moreover, heavy metal (loid)s cannot be degraded after entering the soil, thus causing a long-term cumulative threat. Therefore, various soil-remediation technologies have been developed in recent decades. Conventional soil-remediation techniques usually include physical, chemical, and biological methods, which can be also combined to achieve higher efficiency [1,2]. Among them, phytoremediation has attracted widespread attention due to its green and economic advantages. However, it also has disadvantages, such as low remediation efficiency and time-consuming. Therefore, it is necessary to use other methods to assist phytoremediation.

Due to its high specific surface area, high surface activity and reduction ability, nano-zero-valent iron (nZVI) can effectively remove soil heavy metal(-loid) pollution (e.g., Cd, Cu, Cr, Pb, Zn, and As) [3,4,5]. nZVI can immobilize heavy metals in the soil by adsorption, and the formation of multiple co-precipitations. [5]. It can also reduce the toxicity and mobility of easily reducible heavy metals (such as Cr(VI)) through redox [6,7]. Our previous study found that starch stabilized nZVI (S-nZVI) and bare nZVI (B-nZVI) reduced the concentration of available Cr in soil and subsequently decreased Cr accumulation in mung beans [3]. Low concentrations of nZVI can promote plant growth in contaminated soil and enhance the tolerance of plants to heavy metals; however, high concentrations of nZVI impair the growth of plants, easily causing oxidative damage [8,9]. nZVI can also cause oxidative stress in soil microorganisms [10], but the impact is generally dependent on soil context [11]. More efforts should be made on whether nZVI can be used to assist phytoremediation.

Arbuscular mycorrhizal (AM) fungi widely occur in the soil, which can form symbiosis with the majority of land plants. AM fungi can provide plants with mineral nutrients (e.g., P) and improve their adaptability to environmental stress, including soil contamination [12,13]. Moreover, AM fungi can reduce the bioavailability of soil heavy metals or stabilize heavy metals in plants’ roots to improve the tolerance of plants to heavy metals [13]. A previous field study showed that AM fungi and an iron-containing phyllosilicate amendment jointly benefited the growth of plants in heavy metal-contaminated soil [14]. Gonzalez-Chavez et al. [15] found that the co-precipitation of Cu and Fe on the outer hyphae and cell walls of AM fungi. Putatively, nZVI may have a synergistic interaction with AM fungi to jointly enhance phytoremediation. In addition, nZVI may also be detrimental to the colonization and development of AM fungi, thereby affecting their efficacy. However, few studies have focused on the role of nZVI and AM fungi in soil phytoremediation. Wu et al. [16] found that nZVI (0.5%, *w*/*w*) and AM fungi altered metal(loid) uptake and translocation by maize plants. Therefore, to fill the knowledge gap, it is necessary to study the combined effect of nZVI and AM fungi in the phytoremediation of heavy metal-contaminated soil.

Sweet sorghum is a widely planted biomass energy crop that can be used for bioethanol production. Sweet sorghum can adapt to environmental stresses (e.g., heavy metal contamination) [17]. Previous studies have reported the advantages of sweet sorghum in phytoremediation, such as high accumulation of heavy metals, high growth rate and large amounts of biomass [18,19]. Moreover, as an energy crop, sweet sorghum can prevent heavy metals from entering the food chain, thereby protecting the environment and human health. Our previous research found that sweet sorghum, together with AM fungi, showed potential for phytoremediation of molybdenum-contaminated farmland and revegetation in areas disturbed by molybdenum mines, and the production of biomass in these locations [20]. However, nothing is known about the role of AM fungi and nZVI in assisting heavy metal phytoremediation with sweet sorghum.

We hypothesize that nZVI and AM fungi may synergistically enhance the phytoremediation of heavy metal-contaminated soil with sweet sorghum. Here, for the first time, the effects of a series of concentrations of nZVI (bare or starch stabilized nZVI) and an AM fungus (*Acaulospora mellea* ZZ) were investigated in a multi-metal-contaminated agricultural soil. Our aim is to select the appropriate concentration and type of nZVI to assist sweet sorghum (inoculated with or without AM fungi) for the phytoremediation of heavy metal-contaminated soil.

## 2. Materials and Methods

### 2.1. Soil

The soil was sampled from the surface layer (0–20 cm) of an abandoned paddy field contaminated with Cd, Pb and Zn, which was described in our recent study [21]. The soil was passed through a 2 mm sieve and sterilized at 121 °C for 2 h and then air-dried for plant culture. The physiochemical properties of the soil are shown in Table 1.

### 2.2. AM Inoculum and nZVI

*Acaulospora mellea* ZZ was selected for this experiment. In our previous results, *A. mellea* ZZ significantly enhanced plant growth and tolerance in copper-contaminated soil [22] and saline soil [23]. The AM inoculum was propagated using maize grown in sterile sand [22].

Starch-stabilized nZVI (S-nZVI) is synthesized according to the borohydride reduction method described in our previous study [24]. The average particle size is 69.5 nm, the zeta potential is −14.2 mV, and the average surface area is 46.3 m^2^/g. The scanning electron microscopy (SEM) image, transmission electron microscopy (TEM) image and X-ray diffraction (XRD) pattern of S-nZVI are shown in Figure S1. Bare nZVI (B-nZVI) was purchased from Beijing Deke Daojin Science and Technology Co., Ltd. The basic properties of B-nZVI are as follows: purity 99.9%, average particle size 30–50 nm, and specific surface area 40–60 m^2^/g. The TEM image and XRD pattern are shown in Figure S1.

### 2.3. Experimental Design and Procedure

In order to study the single and combined effects of AM fungi and nZVI, a three-factor experiment was designed, including (a) two inoculation treatments, i.e., inoculation and non-inoculation with AM fungus, *A. mellea* ZZ, (2) two nZVI treatments, i.e., S-nZVI and B-nZVI, and (3) different nZVI concentrations, i.e., 0, 50, 100, 200, 500, and 1000 mg/kg, respectively. Four replicates were set for each treatment. An appropriate amount of each nZVI was mixed into the soil to obtain the target concentration. After that, 1 kg of soil mixture was put into each pot. For the inoculation treatment, 50 g AM inoculum was mixed into each pot. In the non-inoculation treatment, each pot received an equal amount of sterile AM inoculum. A control filtrate of 10 mL AM inoculum was added to all pots to provide similar microbial communities [25].

A widely grown variety (Dalishi) of sweet sorghum was used as the test plant. Twenty surface-sterilized seeds were grown in each pot, and 8 seedlings were retained one week after emergence. The pots were randomly placed in a plant growth chamber at about 25–30 °C with a photoperiod of 12/12 h (light intensity of 10,000 Lux), and a relative humidity of 50–60%. During plant growth, pots were weighed every three days and deionized water was added when necessary to maintain the soil moisture at about 20%.

### 2.4. Sample Analysis

The seedlings were harvested on the 60th day after emergence. Shoots and roots were sampled separately, and the fresh weight was measured after washing with tap water and distilled water. Fresh lateral root samples were taken for root colonization evaluation, based on the procedure described in our previous study [20]. Fresh leaves (0.1 g) were sampled to determine the activities of peroxidase (POD), using the guaiacol method [26], and catalase (CAT), using the colorimetric method [27]. Dried plant tissues were ground and digested using a mixture of acids (HNO_3_:HClO_4_, 4:1, *v/v*) in a graphite digestion instrument (SH220N, Shandong Hanon Instruments Co. Ltd., Jinan, China). ICP-OES (Agilent 720, Agilent Technologies, Santa Clara, CA, U.S.A.) was used to measure the element concentrations of the contaminants Cd, Pb, and Zn, as well as the nutrients Ca, Cu, Fe, Mg, Mn, and P. Diethylenetriaminepentaacetic acid (DTPA) solution (0.005 M DTPA, 0.1 M triethanolamine, 0.01 M CaCl_2_, pH 7.3) was used to extract available metals based on the method described previously [28] and determined using ICP-OES (Agilent 720, Agilent Technologies, Santa Clara, CA, USA). According to the method described by Li et al. [5], the nZVI in the soil received 100 mg/kg nZVI was separated by magnetic separation, and then characterized using an X-ray diffractometer (Rigaku D-Max-2500/PC, Rigaku Industrial Corp., Tokyo, Japan). Samples were scanned from 10 to 90° with CuKα radiation at 40 kV and 150 mA. Energy dispersive X-ray spectroscopy (EDS) and elemental mapping were performed using a Bruker XFlash 6130 energy dispersive spectrometer (Bruker Corp., Karlsruhe, Germany).

### 2.5. Data Analysis

Mycorrhizal response (%) is calculated to estimate the difference in elements absorbed by AM plants relative to non-AM plants (Equation (1)) [29]:

(1) $$\frac{\mathrm{Element}\;\mathrm{content}\;\mathrm{in}\;\mathrm{AM}\;\mathrm{plants}-\mathrm{Element}\;\mathrm{content}\;\mathrm{in}\;\mathrm{non}-\mathrm{AM}\;\mathrm{plants}}{\mathrm{Element}\;\mathrm{content}\;\mathrm{in}\;\mathrm{non}-\mathrm{AM}\;\mathrm{plants}}\times 100$$

Data were submitted to SPSS 22.0 for analysis of variance and significance. The results are expressed as mean ± standard deviation (SD). Duncan’s multiple range test was used to compare the differences between different treatments (*p* < 0.05). Three–way ANOVA was performed to test the main effect and interaction effect between three independent factors (AM inoculation, nZVI type, and nZVI concentration), while two–way ANOVA was tested to analyze the interactions between AM inoculation and each nZVI, or between nZVI type and concentration. The Pearson correlation coefficient was calculated to analyze the correlation between different parameters.

## 3. Results

### 3.1. Root Colonization

**Figure 1:**
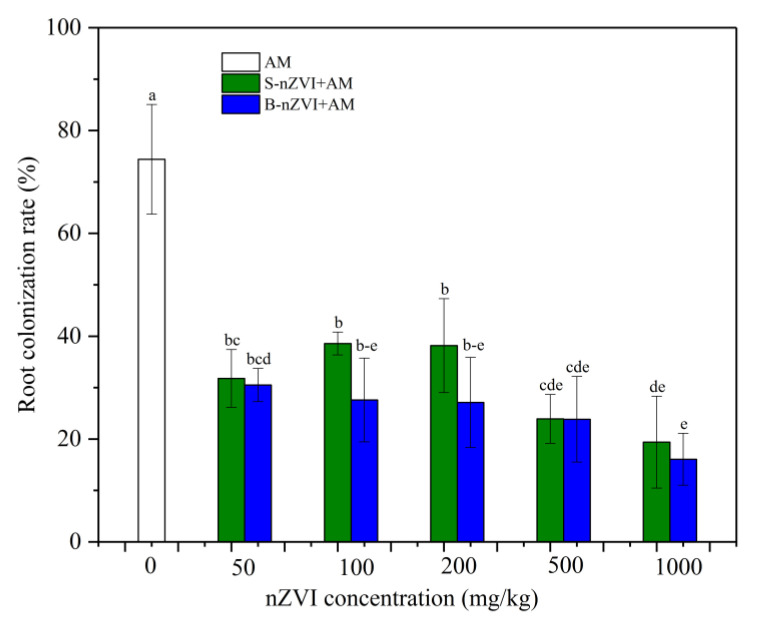
Root mycorrhizal colonization of sweet sorghum plants. Note: AM represents inoculation with *A. mellea* ZZ; S-nZVI+AM represents the treatments that received S-nZVI and AM inoculation; B-nZVI+AM represents the treatments that received B-nZVI and AM inoculation. Different letters above the bars indicate significant differences (*p* < 0.05). Two-way ANOVA results are shown in Table 2.

No mycorrhizal colonization was observed in non-inoculated plants. The AM inoculation successfully colonized the plant roots, with the highest colonization rate of 74.4% in the plants that received no nZVI (Figure 1). The root colonization rate was decreased by both nZVI, displaying a decreasing trend (S-nZVI: −19.4%~−38.6%, B-nZVI: −30%~−16%) with the increase in nZVI concentration. Overall, B-nZVI showed a similar or decreased inhibition effect as compared to S-nZVI. Two-way ANOVA showed that the type and concentration of nZVI had significant impacts, but no significant interaction between them (Table 2).

### 3.2. Pant Biomass

**Figure 2:**
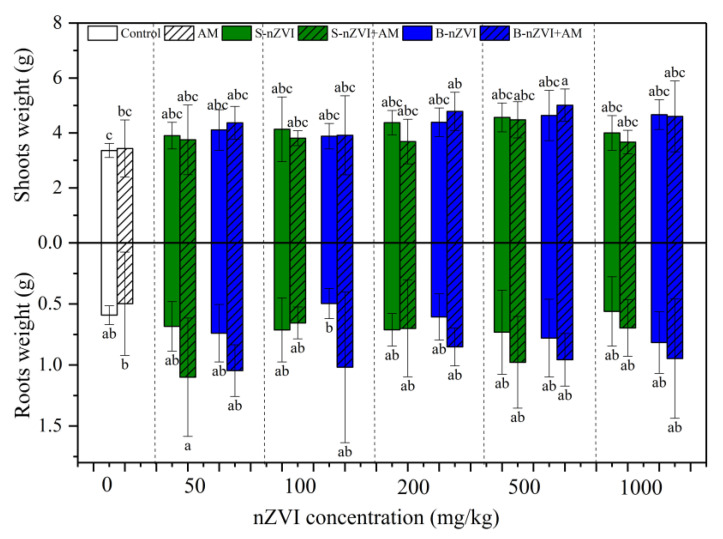
Dry weights of sweet sorghum shoots (above *x*-axis) and roots (below *x*-axis). Control represents the treatment without AM inoculation or nZVI; AM represents the treatments inoculated with *A. mellea* ZZ; S-nZVI represents the treatments that received S-nZVI; S-nZVI+AM represents the treatments that received S-nZVI and AM inoculation; B-nZVI the treatments that received B-nZVI; B-nZVI+AM represents the treatments that received B-nZVI and AM inoculation. Different letters above or below the bars indicate significant differences (*p* < 0.05). Three-way and two-way ANOVA results are shown in Table 2 and Table 3, respectively.

AM inoculation alone did not significantly affect shoot or root dry weights (Figure 2). Although the effects were not significant in most treatments, nZVI had a tendency to increase shoot and root dry weights, particularly in the combination treatments (Figure 2). The inoculated plants that received 500 mg/kg B-nZVI had the highest shoot dry weights, whereas the inoculated plants that received 50 mg/kg S-nZVI had the highest root dry weights. Based on the three-way and two-way ANOVA results, B-nZVI showed significant effects on the shoot dry weight, but there was no significant interaction with the AM inoculation (Table 2 and Table 3).

### 3.3. Bioavailability of Metals in Soil

**Figure 3:**
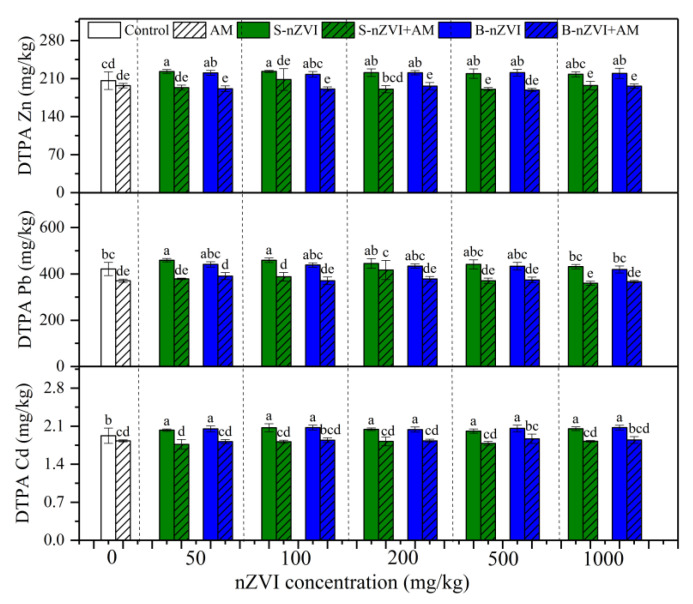
Concentrations of DTPA- Cd, -Pb and -Zn in soil after plant harvest. Control represents the treatment without AM inoculation or nZVI; AM represents the treatments inoculated with *A. mellea* ZZ; S-nZVI represents the treatments that received S-nZVI; S-nZVI+AM represents the treatments that received S-nZVI and AM inoculation; B-nZVI the treatments that received B-nZVI; B-nZVI+AM represents the treatments that received B-nZVI and AM inoculation. Different letters above the bars indicate significant differences (*p* < 0.05). Three-way and two-way ANOVA results are shown in Table 2 and Table 3, respectively.

Irrespective of nZVI, AM inoculation significantly decreased the concentrations of DTPA-Cd, -Pb and -Zn in soil (Figure 3); however, in most cases, a single application of B-nZVI or S-nZVI increased them. No significant interaction was observed between AM inoculation and nZVI (Table 2 and Table 3).

AM inoculation significantly decreased the concentrations of DTPA-Fe and -Cu, increased the concentration of DTPA-Mg, but did not affect the concentration of DTPA-Mn (Figure S2). Overall, both nZVI increased the concentration of DTPA-Fe, but had weak effects on DTPA-Cu, -Mg and -Mn.

### 3.4. Heavy Metal Concentrations and Uptake in Plants

**Figure 4:**
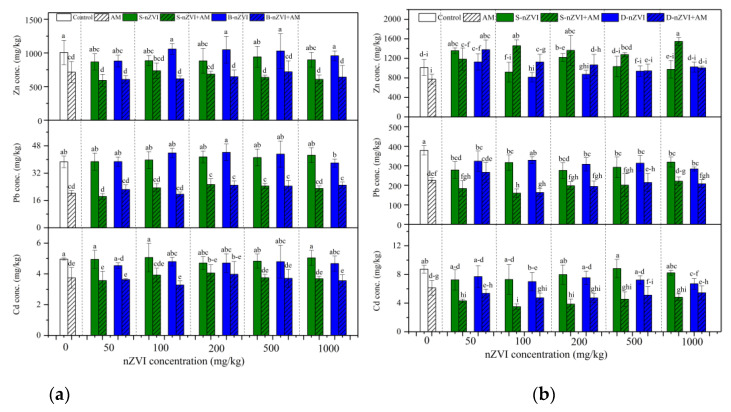
Concentrations of Cd, Pb and Zn in plant shoots (**a**) and roots (**b**). Control represents the treatment without AM inoculation or nZVI; AM represents the treatments inoculated with *A. mellea* ZZ; S-nZVI represents the treatments that received S-nZVI; S-nZVI+AM represents the treatments that received S-nZVI and AM inoculation; B-nZVI the treatments that received B-nZVI; B-nZVI+AM represents the treatments that received B-nZVI and AM inoculation. Different letters above the bars indicate significant differences (*p* < 0.05). Three-way and two-way ANOVA results are shown in Table 2 and Table 3, respectively.

Overall, AM inoculation decreased the concentrations of Cd, Pb, and Zn in shoots and roots, irrespective of nZVI (Figure 4). In most cases, nZVI alone did not affect shoot Cd, Pb and Zn concentrations, decreased root Pb concentrations, and showed an increasing trend in root Zn concentrations. The plants in combination treatments generally had the lowest shoot and root Cd and Pb concentrations. For example, the inoculated plants that received 100 mg/kg S-nZVI had the lowest root Pb and Cd concentrations. The three-way ANOVA results showed significant tripartite interactions among AM inoculation and nZVI type and concentration on shoot Pb and root Zn concentrations (Table 2).

**Figure 5:**
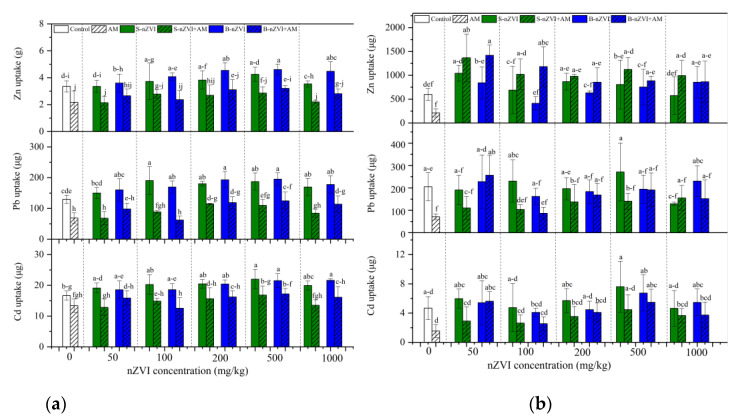
Uptake of Cd, Pb and Zn in plant shoots (**a**) and roots (**b**). Control represents the treatment without AM inoculation or nZVI; AM represents the treatments inoculated with A. mellea ZZ; S-nZVI represents the treatments that received S-nZVI; S-nZVI+AM represents the treatments that received S-nZVI and AM inoculation; B-nZVI the treatments that received B-nZVI; B-nZVI+AM represents the treatments that received B-nZVI and AM inoculation. Different letters above the bars indicate significant differences (*p* < 0.05). Three-way and two-way ANOVA results are shown in Table 2 and Table 3, respectively.

Despite of nZVI, AM inoculation generally decreased shoot Cd, Pb and Zn uptake, and root Cd and Pb uptake (Figure 5). Both nZVI increased shoot Cd, Pb and Zn uptake, and root Zn uptake, but showed weak effects for root Cd and Pb uptake, which varied with nZVI type and concentration. The two-way ANOVA results showed no significant interaction between AM inoculation and S-nZVI or B-nZVI (except for AM inoculation-B-nZVI interaction on root Zn uptake) (Table 3).

### 3.5. XRD, EDS and Mapping Analyses of nZVI

**Figure 6:**
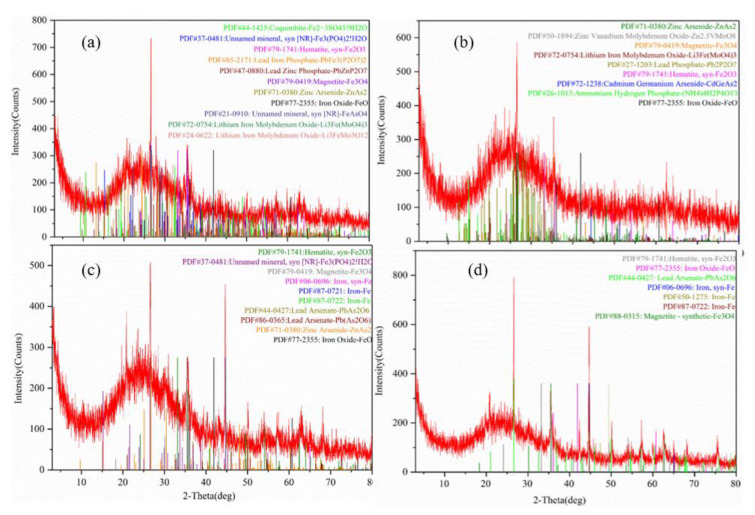
XRD patterns of nZVI after reaction. (**a**) 100 mg/kg S-nZVI; (**b**) 100 mg/kg S-nZVI+AM; (**c**) 100 mg/kg B-nZVI; (**d**) 100 mg/kg B-nZVI+AM.

After plant harvest, Fe^0^ peak was observed in the XRD patterns of B-nZVI but disappeared in the XRD patterns of S-nZVI (Figure 6). There were more heavy metal compounds in S-nZVI, such as PbZnP_2_O_4_, PbFe_3_(P_2_O_7_)_2_, which were not observed in B-nZVI. AM inoculation changed the state of the iron element. For example, the compounds such as Fe_3_(PO_4_)H_2_O, PbZnP_2_O_4_, PbFe_3_(P_2_O_7_)_2_, and ZnAs_2_ were only observed in non-inoculation treatments.

**Figure 7:**
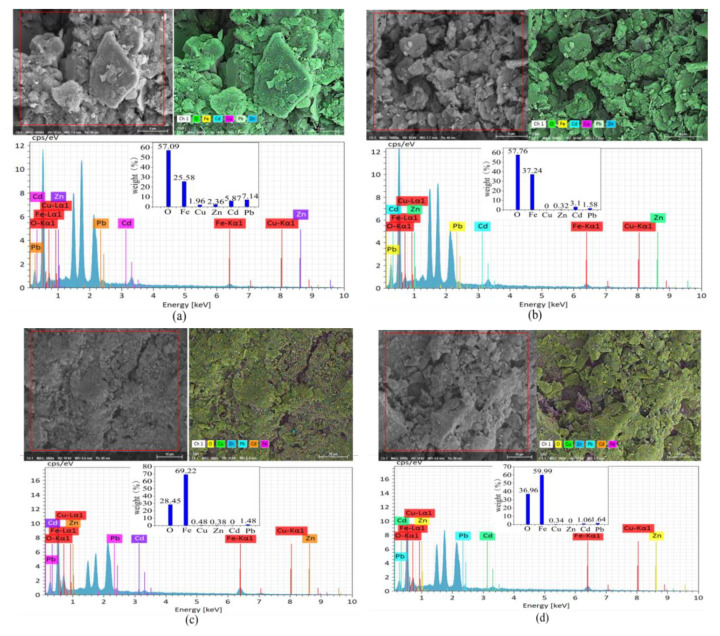
Mapping and EDS analyses of nZVI after reaction. (**a**) 100 mg/kg S-nZVI; (**b**) 100 mg/kg S-nZVI+ AM; (**c**) 100 mg/kg B-nZVI; (**d**) 100 mg/kg B-nZVI+AM.

EDS and mapping analyses confirmed the occurrence of Pb, Cd and/or Zn on the surfaces of S-nZVI and B-nZVI (Figure 7). AM inoculation decreased the relative weight (%) of Pb, Cd and Zn on S-nZVI, but increased the relative weight (%) of Pb and Cd on B-nZVI.

### 3.6. Mycorrhizal Response

Mycorrhizal responses of heavy metal concentrations and uptake in plants are summarized in Table 4. AM inoculation always showed inhibitory effects on shoot and root concentrations and uptake of Cd, Pb and Zn. However, nZVI application changed mycorrhizal responses. For example, the mycorrhizal responses of root Zn concentration and uptake were negative values when no nZVI was applied, whereas in most cases, they shifted to positive values when nZVI was applied.

## 4. Discussion

### 4.1. Effect of AM Fungi on Phytoremediation

In this study, AM inoculation effectively immobilized a variety of heavy metals, evidenced by the decreased metal concentrations in plant roots and shoots (Figure 4). This cannot be ascribed to “growth dilution effects”, because AM inoculation did not increase plant biomass in our present experiment. The first explanation might be due to the retention of AM fungal structures (e.g., extraradical hyphae, and spores) and the chelation of some fungal secretions (e.g., glomalin-related soil protein) [13]. Thus, AM fungi reduced the availability of heavy metals in the soil (Figure 3). Second, in addition to the immobilization of heavy metals in the soil, the AM fungi could bind heavy metals in the fungal structures in the roots (e.g., intraradical hyphae, and vesicles), thereby inhibiting the transport of heavy metals to the aerial parts [16,22,25,30,31]. For example, when nZVI was added, the AM inoculation increased the root Zn concentration, but did not increase the shoot Zn concentration. Third, the AM fungi promoted the absorption of Ca, Mg, and Mn by roots (Figures S3 and S4). The improved mineral nutrients facilitate the plants’ tolerance to heavy metals. The Pearson correlation analyses showed that root Ca, Mg, and Mn concentrations were negatively correlated with root Cd and Pb concentrations (Table S1). These essential nutrients compete with Cd and Pb, thereby inhibiting the absorption of heavy metals by plants [32]. In conclusion, the AM fungi significantly reduced the bioavailability of toxic metals in the soil and subsequently, their accumulation in plants, confirming their contribution to phytostabilization with sweet sorghum, and their potential application in the safe production of crops.

Unexpectedly, AM inoculation did not significantly increase plant biomass, which may be ascribed to several reasons. First, the effects of AM fungi on plant P acquisition from the soil highly vary with soil fertility, particularly the available P [33]. Plant growth promotion by AM fungi is generally maintained in soil with poorly available P [34] but tends to disappear in soil with highly available P [35]. In our present study, the available P is high (27 mg/kg) in the test soil, and plant P nutrition was not improved by AM inoculation (Figure S5). Second, since AM fungi belong to obligate biotrophs that depend on host plants to provide photosynthates for them, their effects on plant growth are not always positive [36]. Mycorrhizal responses are generally more significant for those plant species/cultivars with less tolerance to environmental stress [37,38]. Due to the high tolerance of sweet sorghum to heavy metal toxicity [17], the seedlings in our current experiment exhibited no obvious toxicity symptoms, indicating they did not suffer from the stress caused by the heavy metal(loid)s. Hence, the benefits from AM fungi inoculation may be concealed. Overall, AM fungi alone did not affect the growth of sweet sorghum but decreased heavy metal uptake, implying they cannot be used for heavy metal phytoextraction.

### 4.2. Effect of nZVI on Phytoremediation

Our results show that nZVI, at all the concentrations, had no phytotoxicity but rather promoting effects on plant growth, confirming that nZVI is non-toxic to sweet sorghum. Some previous studies have found that nZVI exhibits “low-concentration promotion and high-concentration inhibition effects” on plant growth [8,9,39]. Our previous experiments found that high concentrations of S-nZVI could be adhered to the root surface and penetrated into roots, decreasing their nutrient uptake and inducing oxidative damage in plants [24], and thus caused higher phytotoxicity than B-nZVI [3]. However, in our present experiment, exposure to nZVI did not increase Fe concentrations in plant shoots and roots (Figures S3 and S4), which indicates that nZVI and the Fe released by nZVI did not enter into the plant tissues. Both S-nZVI and B-nZVI did not disturb nutrient uptake or induce oxidative stress (Figure S6). In addition, the impact of nZVI on plants varies with plant species [40]. Sweet sorghum is considered a plant with strong tolerance to environmental stress [41]. In general, nZVI is lowly toxic or non-toxic to sweet sorghum, indicating potential combined applications of nanoremediation and phytoremediation.

B-nZVI and S-nZVI showed different effects on heavy metal accumulation in plants, which vary depending on nZVI type and concentration, and the mycorrhizal status. In most cases, both nZVI alone did not influence shoot Zn, Cd and Pb concentrations (Figure 4a) but sometimes enhanced their uptake in the shoots of non-inoculated plants (Figure 5a). These results imply the potential use of nZVI in phytoextraction. In non-mycorrhizal plants, 1000 mg/kg B-nZVI significantly reduced root Cd concentration compared to the control (Figure 4), whereas in mycorrhizal plants, 100 mg/kg S-nZVI greatly inhibited root Cd accumulation (Figure 4). Moreover, in mycorrhizal plants, S-nZVI treatments increased root Zn concentrations, which benefits the root immobilization of Zn. nZVI can immobilize metals such as Cd, Pb, and Zn in the soil mainly through adsorption, co-precipitation, and the formation of multiple complexes [5,42,43]. Although nZVI did not influence the DTPA-extractability of Cd, Pb and Zn, the XRD patterns confirmed more compounds (particularly Pb) on the surface of reacted nZVI, obtained by magnetic separation (Figure 6). We observed a decrease in Pb concentration in the roots treated with S-nZVI or B-nZVI (Figure 4). The Pearson correlation analysis showed significantly positive correlation between the concentrations of Fe and Pb in the roots (Table S1), indicating that the iron-containing compound formed by nZVI (or its derived oxide) on the root surface was involved in the precipitation of Pb [16].

The ability of nZVI to immobilize metal(loid)s depends to a large extent on the characteristics of the metal(loid)s [4]. There might be competition among the metal(loid)s. For example, the presence of Pb and Zn in the soil would reduce the ability of nZVI to immobilize Cd [4]. nZVI would preferentially remove metal(loid)s with a standard electrode potential (E^0^) larger than Fe (e.g., As, Cr, Cu, and Hg) through reduction and precipitation, while metal(loid)s with E^0^ smaller than Fe (e.g., Zn and Cd) cannot be reduced, but only sorbed or complexed [4,44]. Pb has E^0^ slightly larger than nZVI and can be immobilized by nZVI via sorption and precipitation, which may partly explain why nZVI decreased root Pb concentration but did not decrease Zn and Cd accumulation (Figure 5).

Soil pH influences the immobilization of metal(loid)s by nZVI [4]. The pH value affects the surface isoelectric point of nZVI, which subsequently affects its affinity for metal ions [45]. At higher pH values, more oxygen-containing groups (e.g., oxides or oxyhydroxides) in reacted nZVI were deprotonated and the surface was negatively charged, which facilitates the adsorption of more heavy metal ions through electrostatic interactions [46]. The soil in our experiment was acidic (pH = 5.0), and a lower pH may reduce the ability of nZVI (and the derived oxide or hydroxide) to immobilize Pb, Zn, and Cd [44].

### 4.3. AM Fungi and nZVI Interactions on Phytoremediation

AM colonization rates can reach higher than 70%, indicating the tolerance of *A. melllea* ZZ to the combined pollution by Cd, Pb and Zn. This is similar to our previous findings that *A. melllea* ZZ is tolerant to various pollutants [22,47,48]. Furthermore, we found that nZVI inhibited AM colonization, depending mainly on nZVI concentration. Many nanoparticles, such as Ag, FeO, and nZVI, have been shown toxic to AM fungi [11,49,50]. Previous studies have found that nZVI was not conducive to AM colonization in roots [16,51], and reduced the biomass of AM fungi in the soil [11]. Possible reasons may include the following: (1) nZVI may cause cell membrane disruption and oxidative damage through the generation of Fe^2+^ and reactive oxygen species [52,53], and thus directly impair AM fungi, and (2) nZVI may cause oxidative damage in plants and thus indirectly inhibit AM colonization in plant roots [16]. Here, our results confirm the fungitoxicity of both B-nZVI and S-nZVI. Given that AM fungi are a group of symbiotic microbes with important ecological functions, the impacts of nZVI on AM fungal community composition and function should be taken into account when nZVI is applied in soil remediation.

Despite nZVI type and concentration, the AM colonization rate of inoculated plants reached as high as 20%, even in the roots exposed to 1000 mg/kg nZVI, indicating a high tolerance of *A. melllea* ZZ to nZVI toxicity, and their combined applications. Both AM fungi and appropriate nZVI can benefit plant nutrient uptake and growth, and safe crop production in contaminated soils [12,13,54,55]. It is necessary to select and introduce tolerant AM fungal strains to encounter nZVI toxicity during the nanoremediation program.

Irrespective of the inhibited AM colonization by nZVI, we found some synergistic effects of nZVI and AM inoculation on metal immobilization. For example, the plants in combination treatments generally had the lowest root Pb and Cd concentrations (Figure 4). In fact, nZVI substantially changed mycorrhizal responses of contaminant accumulation (Table 4). In turn, AM fungi may facilitate nZVI (or derived oxide) to form Fe compounds on the root surface [16], which might be involved in the precipitation of toxic metals [56]. AM fungi may accelerate the collapse and corrosion of nZVI, making the surface of nZVI looser and more porous (Figure 7), which subsequently increases the adsorption capacity of nZVI (or derived oxides) and the contact area with pollutants. On the other hand, AM fungi may counteract metal(loid) immobilization by nZVI [16]. For example, AM fungi might directly interact with nZVI, and dissolve the heavy metals stabilized by nZVI, thus reducing the efficiency of nZVI immobilization [16]. Hence, the interactions of AM fungi and nZVI on heavy metal immobilization can vary from synergistic to antagonistic, which need further investigation.

More importantly, nZVI can induce phytotoxicity even at environmentally relevant concentrations [39,57,58,59]. Plants exposed to nZVI might accumulate too many Fe ions released by nZVI, thereby disrupting the absorption and transportation of nutrients and the nutritional balance in plant tissues [24,60]. Our results showed that AM fungi decreased the availability of the Fe released by nZVI and subsequent uptake by roots, thereby reducing the potential ecological risk of nZVI. Furthermore, AM inoculation reduced nZVI inhibition on the water uptake of maize plants [16] and alleviated the physiological stresses in white willow caused by a high dose of nZVI [51]. AM fungi help plants overcome heavy metal toxicity and grow better, which is a prerequisite for the phytoremediation of heavy metal-contaminated soil. The introduction of rhizosphere microorganisms, such as AM fungi, can intensify the positive effect and neutralize the adverse effect of nZVI [51]. Overall, the combined use of mycorrhizal sweet sorghum and nZVI may be a feasible technique to assist the phytostabilization of heavy metals in contaminated soils.

## 5. Conclusions

We tested the single and joint effects of AM fungus and nZVI (S-nZVI and B-nZVI) on the phytoremediation of an acidic soil with realistic environmental pollution, using sweet sorghum. We found that the remediation effects depended on AM fungi and the type and dosage of nZVI. AM inoculation alone effectively immobilized heavy metals in soil and decreased heavy metal accumulation in plants. Both S-nZVI and B-nZVI at all test concentrations (50–1000 mg/kg) showed no phytotoxicity to sweet sorghum, and enhanced the phytoextraction of heavy metals and the immobilization of heavy metals (particularly Pb) on their surface. AM inoculation successfully colonized plant roots, irrespective of the fungitoxicity of nZVI. Overall, low concentrations of nZVI (e.g., 100 mg/kg) and AM inoculation had a synergistic effect on heavy metal immobilization, indicating their combined applications in the future phytoremediation of heavy metal-contaminated soil with sweet sorghum.

## Figures and Tables

**Table 1:** The physiochemical properties of the soil.

Item	Value
pH	5.0
Total Cd	2.6 mg/kg
Total Pb	1796 mg/kg
Total Zn	1603 mg/kg
DTPA-Cd	1.90 mg/kg
DTPA-Pb	412.6 mg/kg
DTPA-Zn	237.9 mg/kg
DTPA-Cu	7.5 mg/kg
DTPA-Mn	68.5 mg/kg
DTPA-Fe	154.6 mg/kg
DTPA-Mg	84.2 mg/kg
Organic matter	25.8 g/kg
Available P	27 mg/kg
Available K	26.3 mg/kg
Available N	118 mg/kg
Cation exchange capacity	3.15 cmol/kg
Soil type	Paddy soil

**Table 2:** Significance levels (*F* values) of AM inoculation, nZVI type and concentration, and their interactions on measured parameters on a three-way ANOVA analysis.

Variable	AM	nZVI Type	nZVI Conc.	AM×Type	AM×Conc.	Types ×Dose	AM×Type×Conc.
Colonization rate	-	6.75 ***	5.37 *	-	-	1.06 NS	-
Root biomass	3.32 NS	0.58 NS	0.84 NS	0.43 NS	0.44 NS	0.63 NS	1.03 NS
Shoot biomass	0.00 NS	4.36 *	2.03 NS	1.07 NS	0.12 NS	0.62 NS	0.08 NS
DTPA-Cd	225.02 ***	5.03 *	1.03 NS	0.64 NS	0.38 NS	0.70 NS	0.04 NS
DTPA-Pb	245.21 ***	8.39 **	6.36 ***	0.99 NS	1.78 NS	1.77 NS	1.78 NS
DTPA-Zn	155.19 ***	1.34 NS	1.29 NS	0.37 NS	0.54 NS	1.73 NS	1.39 NS
DTPA-Fe	349.83 ***	17.05 ***	19.91 ***	4.53 *	3.70 **	3.81 **	2.02 NS
DTPA-Cu	614.76 ***	2.16 NS	0.87 NS	0.81 NS	0.13 NS	1.26 NS	0.28 NS
DTPA-Mn	4.95 *	7.36 **	2.05 NS	1.62 NS	0.51 NS	0.81 NS	0.84 NS
DTPA-Mg	252.83 ***	1.45 NS	0.68 NS	0.74 NS	0.87 NS	0.69 NS	0.79 NS
Shoot Cd conc.	81.53 ***	2.45 NS	0.25 NS	0.06 NS	0.85 NS	0.42 NS	0.38 NS
Shoot Pb conc.	447.99 ***	0.73 NS	3.44 *	0.04 NS	0.58 NS	0.48 NS	2.79 *
Shoot Zn conc.	96.16 ***	2.10 NS	1.64 NS	3.94 NS	0.01 NS	0.25 NS	0.94 NS
Shoot Fe conc.	0.04 NS	10.94 **	0.75 NS	29.82 ***	0.40 NS	0.70 NS	3.38 *
Shoot Ca conc.	22.18 ***	5.65 *	0.58 NS	4.18 *	1.54 NS	6.41 *	0.74 NS
Shoot Cu conc,	81.02 ***	3.46 NS	0.52 NS	6.61 *	0.34 NS	1.01 NS	0.35 NS
Shoot Mn conc.	73.18 ***	0.16 NS	2.79 *	4.94 *	1.50 NS	0.85 NS	0.70 NS
Shoot Mg conc.	19.45 ***	2.98 NS	1.70 NS	0.08 NS	0.30 NS	0.73 NS	1.52 NS
Shoot P conc.	22.96 ***	0.11 NS	0.97 NS	0.60 NS	0.21 NS	1.06 NS	0.49 NS
Root Cd conc.	122.17 ***	0.13 NS	1.29 NS	10.34 **	0.73 NS	1.07 NS	0.37 NS
Root Pb conc.	164.67 ***	3.56 NS	0.95 NS	0.00 NS	3.45 *	2.78 *	0.55 NS
Root Zn conc.	8.41 **	21.94 ***	5.19 **	0.73 NS	2.28 NS	2.57 *	5.22 **
Root Fe conc.	212.21 ***	10.17 **	1.08 NS	0.27 NS	2.34 NS	1.82 NS	1.02 NS
Root Ca conc.	68.51 ***	2.71 NS	3.36 *	11.48 **	3.09 *	2.08 NS	1.03 NS
Root Cu conc.	236.01 ***	3.19 NS	4.40 **	0.00 NS	1.23 NS	3.95 **	0.83 NS
Root Mn conc.	162.02 ***	3.56 NS	3.40 *	4.49 *	1.10 NS	3.02 *	3.01 *
Root Mg conc.	33.62 ***	0.00 NS	6.32 ***	4.48 **	1.21 NS	3.05 *	1.23 NS
Root P conc.	145.40 ***	0.08 NS	4.07 **	7.07 *	0.59 NS	2.70 *	4.12 **
Shoot Cd uptake	67.38 ***	0.35 NS	3.79 **	0.99 NS	0.34 NS	1.70 NS	0.42 NS
Shoot Pb uptake	183.87 ***	1.74 NS	6.67 ***	0.41 NS	1.41 NS	2.18 NS	0.35 NS
Shoot Zn uptake	85.16 ***	9.07 **	3.97 **	0.69 NS	0.27 NS	0.97 NS	0.41 NS
Root Cd uptake	16.64 ***	0.14 NS	3.22 *	2.17 NS	0.14 NS	0.35 NS	0.55 NS
Root Pb uptake	16.79 ***	1.33 NS	1.47 NS	1.95 NS	0.79 NS	2.15 NS	1.54 NS
Root Zn uptake	4.87 *	1.05 *	3.01 *	0.08 NS	1.02 NS	0.36 NS	1.08 NS
CAT	5.64 *	0.03 NS	1.84 NS	1.72 NS	1.18 NS	0.19 NS	0.72 NS
POD	5.51 *	7.26 **	1.83 NS	0.02 NS	2.74 *	0.91 NS	1.27 NS

Significance levels: * *p* < 0.05, ** *p* < 0.01, *** *p* < 0.001, NS: Non-significance.

**Table 3:** Significance levels (*F* values) of AM inoculation, nZVI, and their interactions on measured parameters on a two-way ANOVA analysis.

Variable	S-nZVI			B-nZVI		
	AM	Conc.	AM×Conc.	AM	Conc.	AM×Conc.
Root biomass	1.43 NS	1.49 NS	0.80 NS	3.85 NS	1.52 NS	0.92 NS
Shoot biomass	0.92 NS	1.82 NS	0.12 NS	0.53 NS	3.00 *	0.10 NS
DTPA-Cd	145.18 ***	1.50 NS	1.97 NS	117.76 ***	2.11 NS	1.42 NS
DTPA-Pb	133.42 ***	5.09 **	2.12 NS	181.83 ***	2.54 *	0.42 NS
DTPA-Zn	74.47 ***	2.17 NS	1.95 NS	131.76 ***	1.17 NS	2.29 NS
DTPA-Fe	150.72 ***	9.38 ***	2.19 NS	213.99 ***	20.10 ***	1.64 NS
DTPA-Cu	276.96 ***	2.13 NS	0.74 NS	242.35 ***	2.20 NS	0.39 NS
DTPA-Mn	0.97 NS	1.49 NS	0.54 NS	9.43 **	1.14 NS	0.96 NS
DTPA-Mg	186.55 ***	2.13 NS	0.41 NS	123.03 ***	2.66 *	0.84 NS
Shoot Cd conc.	58.01 ***	0.20 NS	0.54 NS	47.65 ***	0.48 NS	0.50 NS
Shoot Pb conc.	305.09 ***	2.43 NS	0.56 NS	308.35 ***	2.16 NS	2.20 NS
Shoot Zn conc.	49.81 ***	1.07 NS	0.94 NS	66.68 ***	1.05 NS	0.37 NS
Shoot Fe conc.	12.00 **	0.50 NS	2.81 *	13.65 **	1.51 NS	1.80 NS
Shoot Ca conc.	5.77 *	5.10 **	0.54 NS	28.18 ***	5.43 **	1.74 NS
Shoot Cu conc.	34.62 ***	0.64 NS	0.95 NS	65.14 ***	0.78 NS	0.41 NS
Shoot Mn conc.	25.62 ***	2.65 *	0.97 NS	68.42 ***	3.29 *	0.85 NS
Shoot Mg conc.	11.25 **	0.96 NS	1.41 NS	10.30 **	2.18 NS	0.50 NS
Shoot P conc.	11.41 **	1.02 NS	0.52 NS	17.80 **	0.98 NS	0.10 NS
Root Cd conc.	104.52 ***	2.88 *	0.61 NS	63.76 ***	2.37 NS	0.65 NS
Root Pb conc.	102.97 ***	3.68 **	1.60 NS	125.51 ***	3.93 **	2.89 *
Root Zn conc.	8.45 **	5.24 **	5.71 **	6.10 *	9.35 ***	5.34 **
Root Fe conc.	131.67 ***	5.81 **	2.75 *	138.39 ***	2.72 *	0.81 NS
Root Ca conc.	70.48 ***	2.97 *	0.71 NS	25.13 ***	1.48 NS	3.76 **
Root Cu conc.	143.91 ***	9.24 ***	0.74 NS	140.62 ***	6.77 ***	0.96 NS
Root Mn conc.	124.93 ***	6.11 ***	1.92 NS	101.28 ***	5.15 **	2.80 *
Root Mg conc.	32.41 ***	5.35 **	2.54 *	18.52 ***	1.09 NS	2.50 NS
Root P conc.	94.11 ***	6.66 ***	4.46 **	94.64 ***	0.92 NS	1.54 NS
Shoot Cd uptake	57.37 ***	3.29 *	0.42 NS	39.69 ***	4.28 **	0.58 NS
Shoot Pb uptake	157.45 ***	7.09 ***	0.86 NS	106.13 ***	7.25 ***	0.97 NS
Shoot Zn uptake	42.60 ***	2.13 NS	0.13 NS	68.17 ***	4.69 **	0.51 NS
Root Cd uptake	15.40 ***	1.70 NS	0.33 NS	5.99 *	3.12 *	0.84 NS
Root Pb uptake	18.05 ***	0.97 NS	1.63 NS	4.75 *	2.79 *	1.36 NS
Root Zn uptake	3.42 NS	4.17 **	1.43 NS	6.61 *	5.02 **	3.78 **
CAT	0.67 NS	0.86 NS	2.02 NS	6.10 *	1.24 NS	2.05 NS
POD	3.93 NS	1.13 NS	0.83 NS	2.88 NS	1.66 NS	2.69 *

Significance levels: * *p* < 0.05, ** *p* < 0.01, *** *p* < 0.001, NS: Non-significance.

**Table 4:** Mycorrhizal response (%) as influenced by nZVI.

Parameter		No nZVI	50		100		200		500		1000	
			S-nZVI	B-nZVI	S-nZVI	B-nZVI	S-nZVI	B-nZVI	S-nZVI	B-nZVI	S-nZVI	B-nZVI
Cd conc.	Shoot	−25	−28	−20	−23	−32	−14	−22	−22	−23	−27	−24
	Root	−30	−40	−31	−52	−32	−52	−37	−49	−29	−41	−26
Cd uptake	Shoot	−20	−33	−27	−24	−23	−32	−14	−32	−21	−20	−25
	Root	−67	−51	−45	−38	−41	−21	4	−38	−9	−19	−32
Pb conc.	Shoot	−52	−53	−42	−41	−55	−39	−44	−40	−43	−46	−34
	Root	−40	−34	−18	−50	−50	−28	−37	−31	−32	−30	−27
Pb uptake	Shoot	−55	−55	−39	−53	−54	−45	−38	−41	−47	−50	−36
	Root	−65	−42	−55	−30	−48	20	13	−47	−8	−2	−34
Zn conc.	Shoot	−29	−32	−31	−23	−42	−22	−38	−32	−30	−32	−33
	Root	−24	−13	32	59	38	12	22	24	1	44	−2
Zn uptake	Shoot	−35	−36	−25	−30	−33	−38	−26	−42	−32	−30	−37
	Root	−65	31	48	13	39	73	68	186	35	17	1

## Data Availability

The data presented in this study are available on request from the corresponding author.

## Supplementary Materials

The following are available online at https://www.mdpi.com/article/10.3390/nano11051264/s1, Figure S1: Characterization of two types of nZVI, Figure S2: Concentrations of DTPA-Cu, -Mg, -Mn and -Fe in soil after plant harvest, Figure S3: Concentrations of Fe, Mg, Mn, Cu and Ca in plant shoots, Figure S4: Concentrations of Fe, Mg, Mn, Cu and Ca in plant roots, Figure S5: Concentrations of P in plant shoots (above x-axis) and roots (below x-axis), Table S1: Pearson correlation coefficient between mineral concentration and heavy metal concentration in shoots and roots of sweet sorghum.
