# Supplementary material for: Contribution of Nano-Zero-Valent Iron and Arbuscular Mycorrhizal Fungi to Phytoremediation of Heavy Metal-Contaminated Soil
Source: Nanomaterials (Basel). 2021 May 11;11(5):1264. doi: 10.3390/nano11051264 (PMC8151622; doi:10.3390/nano11051264)
Supplement: Supplementary file 1 [file nanomaterials-11-01264-s001.zip › nanomaterials-1160861-supplementary.pdf]

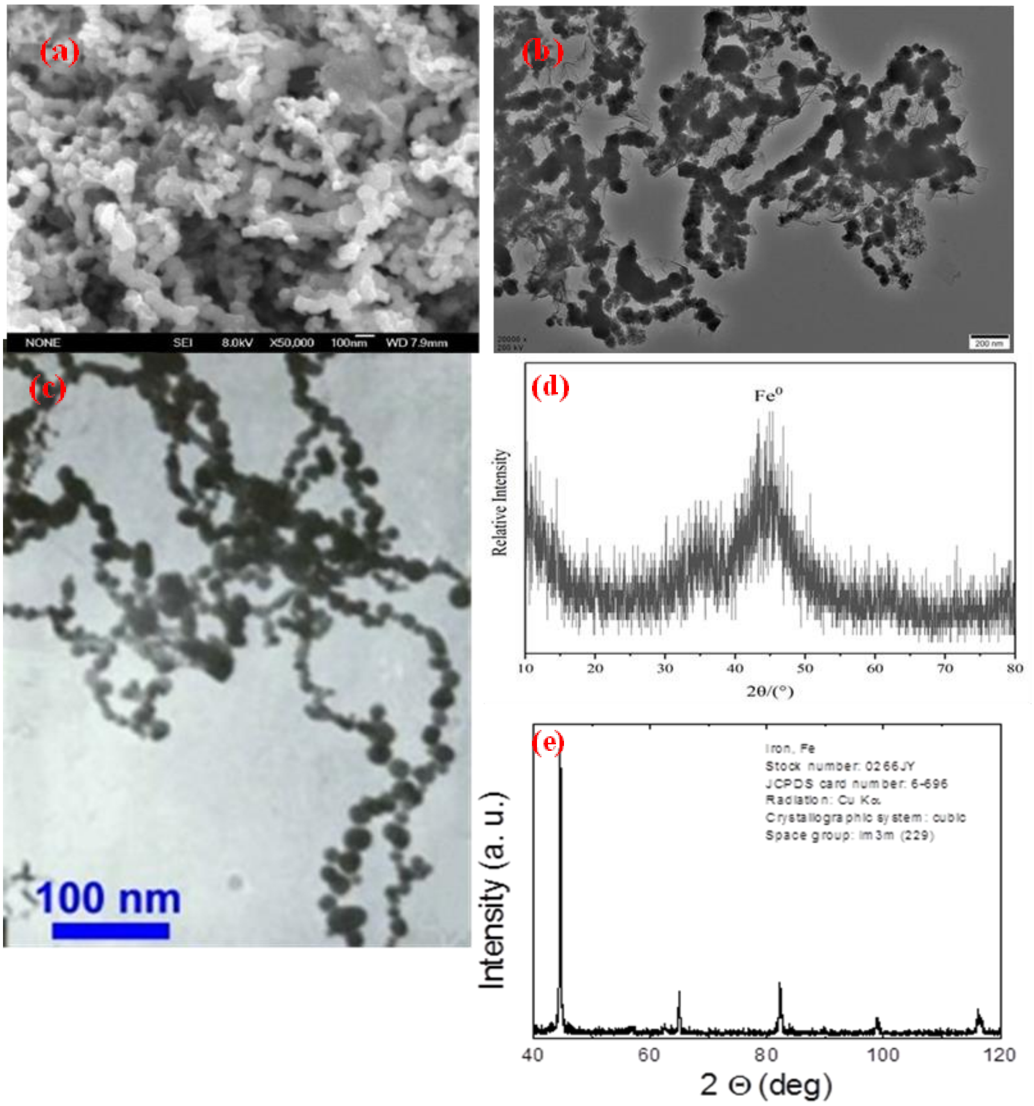

Figure S1. Characterization of two types of nZVI
(a) SEM image of S-nZVI; (b) TEM image of S-nZVI; (c) TEM image of B-nZVI; (d) XRD pattern of S-nZVI; (e) XRD pattern of B-nZVI.

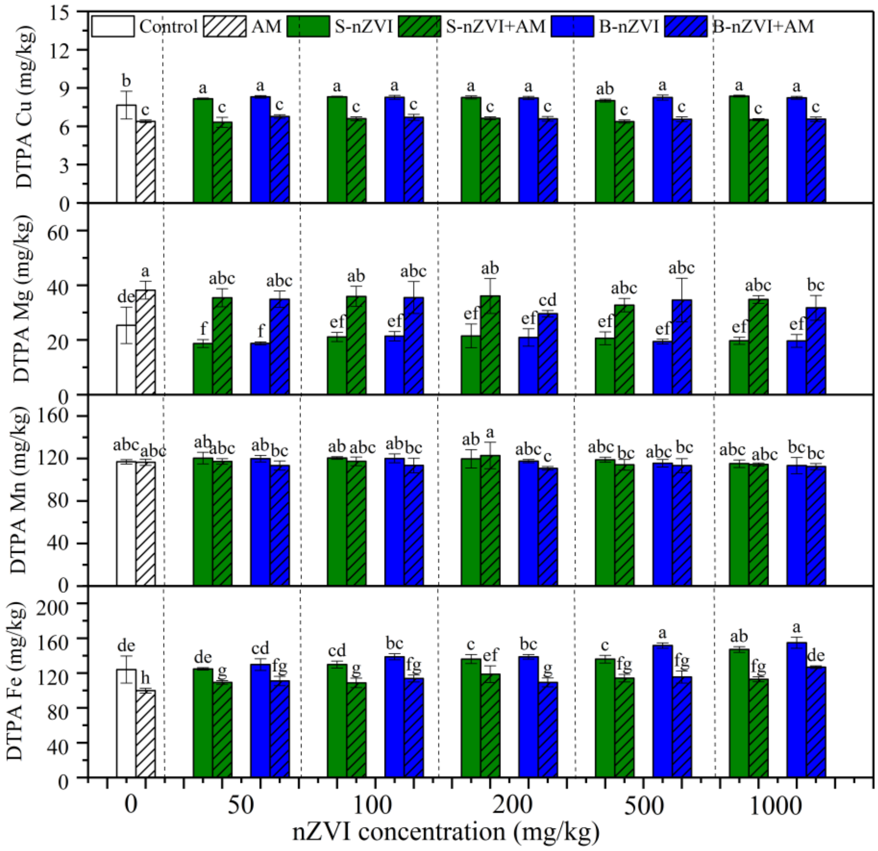

Figure S2. Concentrations of DTPA- Cu, -Mg, -Mn and -Fe in soil after plant harvest. Control represents the treatment without AM inoculation or nZVI; AM represents the treatments inoculated with *A. mellea* ZZ; S-nZVI represents the treatments that received S-nZVI; S-nZVI+AM represents the treatments that received S-nZVI and AM inoculation; B-nZVI the treatments that received B-nZVI; B-nZVI+AM represents the treatments that received B-nZVI and AM inoculation. Different letters above the bars indicate significant differences ($p < 0.05$). Three-way and two-way ANOVA results are shown in Table 2 and Table 3, respectively.

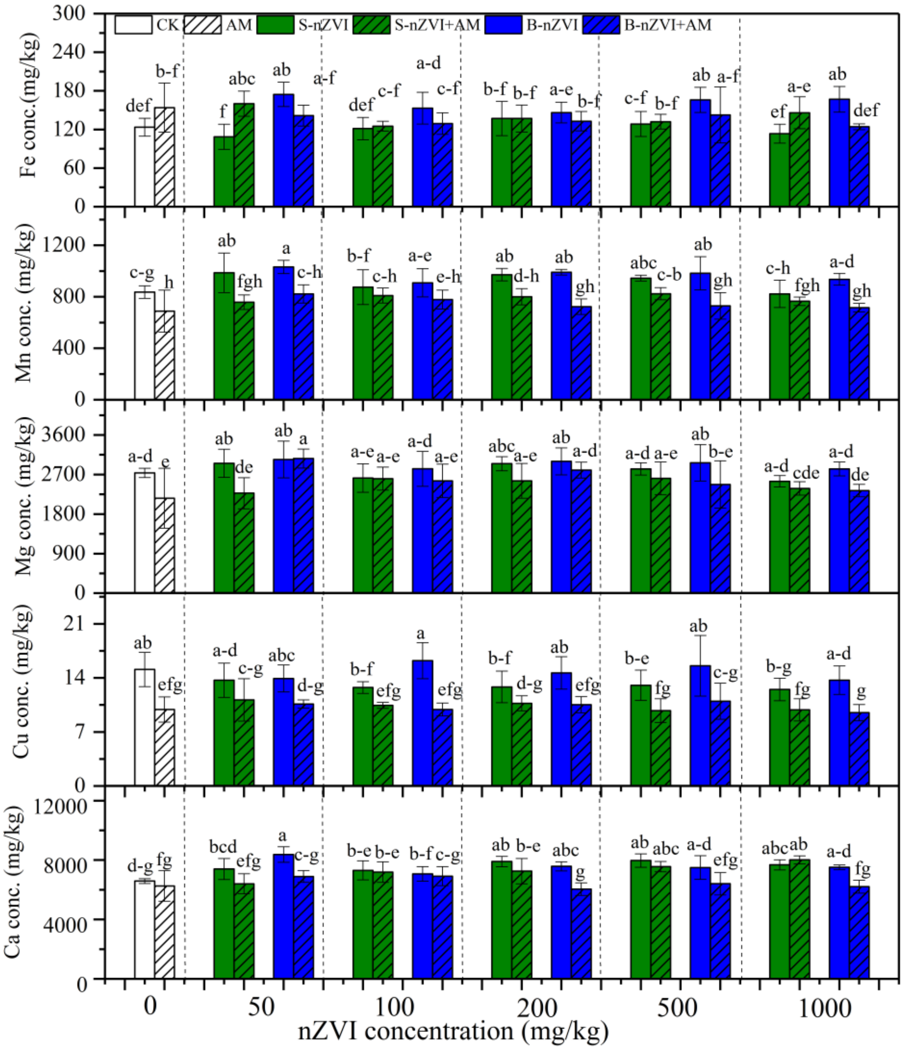

Figure S3. Concentrations of Fe, Mg, Mn, Cu and Ca in plant shoots. Control represents the treatment without AM inoculation or nZVI; AM represents the treatments inoculated with *A. mellea* ZZ; S-nZVI represents the treatments that received S-nZVI; S-nZVI+AM represents the treatments that received S-nZVI and AM inoculation; B-nZVI the treatments that received B-nZVI; B-nZVI+AM represents the treatments that received B-nZVI and AM inoculation. Different letters above the bars indicate significant differences ($p < 0.05$). Three-way and two-way ANOVA results are shown in Table 2 and Table 3, respectively.

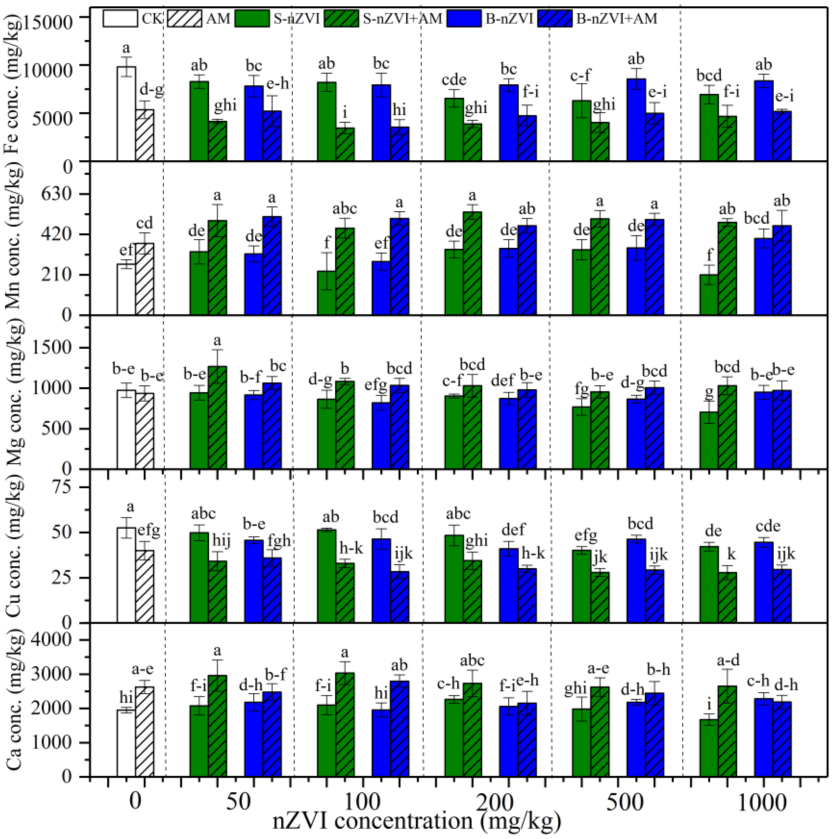

Figure S4. Concentrations of Fe, Mg, Mn, Cu and Ca in plant roots. Control represents the treatment without AM inoculation or nZVI; AM represents the treatments inoculated with *A. mellea* ZZ; S-nZVI represents the treatments that received S-nZVI; S-nZVI+AM represents the treatments that received S-nZVI and AM inoculation; B-nZVI the treatments that received B-nZVI; B-nZVI+AM represents the treatments that received B-nZVI and AM inoculation. Different letters above the bars indicate significant differences ($p < 0.05$). Three-way and two-way ANOVA results are shown in Table 2 and Table 3, respectively.

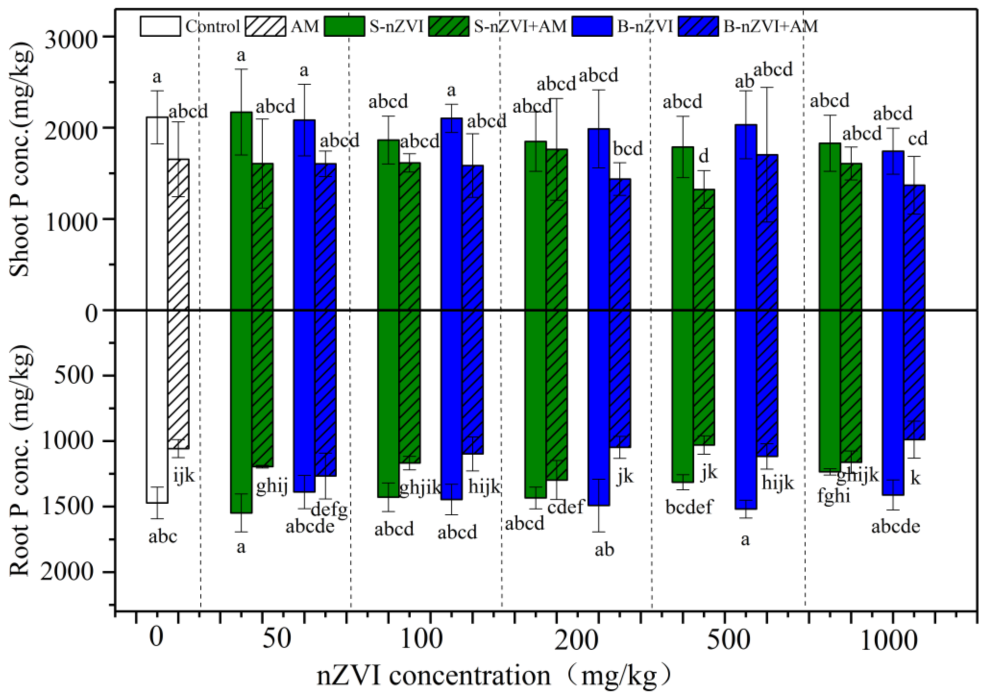

Figure S5. Concentrations of P in plant shoots (above x-axis) and roots (below x-axis). Control represents the treatment without AM inoculation or nZVI; AM represents the treatments inoculated with *A. mellea* ZZ; S-nZVI represents the treatments that received S-nZVI; S-nZVI+AM represents the treatments that received S-nZVI and AM inoculation; B-nZVI the treatments that received B-nZVI; B-nZVI+AM represents the treatments that received B-nZVI and AM inoculation. Different letters above or below the bars indicate significant differences ($p < 0.05$). Three-way and two-way ANOVA results are shown in Table 2 and Table 3, respectively.

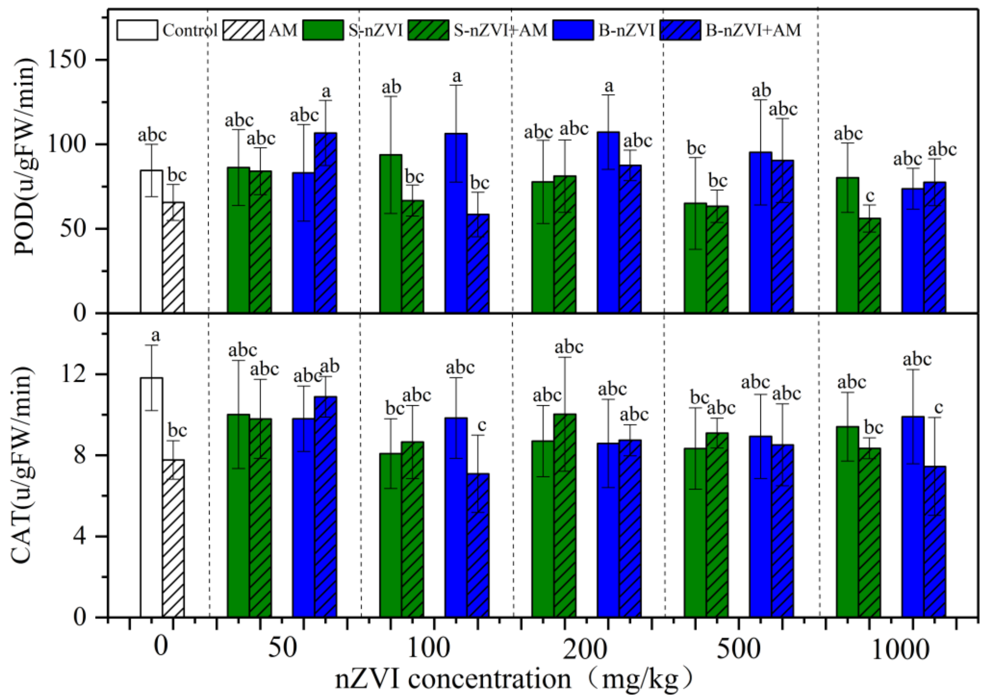

Figure S6. Activity of POD (a) and CAT (b) in plant shoots. Control represents the treatment without AM inoculation or nZVI; AM represents the treatments inoculated with *A. mellea* ZZ; S-nZVI represents the treatments that received S-nZVI; S-nZVI+AM represents the treatments that received S-nZVI and AM inoculation; B-nZVI the treatments that received B-nZVI; B-nZVI+AM represents the treatments that received B-nZVI and AM inoculation. Different letters above the bars indicate significant differences ($p < 0.05$). Three-way and two-way ANOVA results are shown in Table 2 and Table 3, respectively.

Table S1. Pearson correlation coefficient between mineral concentration and heavy metal concentration in shoots and roots of sweet sorghum.

	Cd		Pb		Zn	
	Shoots	Roots	Shoots	Roots	Shoots	Roots
Shoot Ca conc.	0.483*	0.418 NS	0.554**	0.393 NS	0.409 NS	0.279 NS
Shoot Fe conc.	-0.074NS	-0.040 NS	0.065 NS	0.109 NS	0.153 NS	-0.127 NS
Shoot Mg conc.	0.554**	0.486*	0.643**	0.558**	0.565**	0.040 NS
Shoot Mn conc.	0.736**	0.656**	0.822**	0.657**	0.753**	-0.058 NS
Root Ca conc.	-0.785**	-0.843**	-0.821**	-0.820**	-0.717**	0.536*
Root Fe conc.	0.864**	0.855**	0.861**	0.936**	0.877**	-0.478*
Root Mg conc.	-0.721**	-0.752**	-0.782**	-0.658**	-0.694**	0.520*
Root Mn conc.	-0.884**	-0.869**	-0.839**	-0.846**	-0.811**	0.525*

Significance levels: * $p < 0.05$, ** $p < 0.01$, NS Non-significance.
